# How Low Nucleation Density of Graphene on CuNi Alloy is Achieved

**DOI:** 10.1002/advs.201700961

**Published:** 2018-03-12

**Authors:** Yifan Liu, Tianru Wu, Yuling Yin, Xuefu Zhang, Qingkai Yu, Debra J. Searles, Feng Ding, Qinghong Yuan, Xiaoming Xie

**Affiliations:** ^1^ State Key Laboratory of Precision Spectroscopy School of Physics and Material Science East China Normal University 3663 N. Zhongshan Road Shanghai 200062 China; ^2^ State Key Laboratory of Functional Materials for Informatics Shanghai Institute of Microsystem and information Technology Chinese Academy of Sciences 865 Changning Road Shanghai 200050 China; ^3^ Ingram School of Engineering, and MSEC Texas State University San Marcos TX 78666 USA; ^4^ Centre for Theoretical and Computational Molecular Science Australian Institute for Bioengineering and Nanotechnology and School of Chemistry and Molecular Biosciences The University of Queensland Brisbane QLD 4072 Australia; ^5^ Institute of Textiles and Clothing Hong Kong Polytechnic University Hong Kong S.A.R. 999077 China; ^6^ Center for Multidimensional Carbon Materials Institute for Basic Science School of Materials Science and Engineering Ulsan National Institute of Science and Technology Ulsan 689‐798 South Korea

**Keywords:** carbon diffusion, CuNi alloys, graphene nucleation, percolation

## Abstract

CuNi alloy foils are demonstrated to be one of the best substrates for synthesizing large area single‐crystalline graphene because a very fast growth rate and low nucleation density can be simultaneously achieved. The fast growth rate is understood to be due the abundance of carbon precursor supply, as a result of the high catalytic activity of Ni atoms. However, a theoretical understanding of the low nucleation density remains controversial because it is known that a high carbon precursor concentration on the surface normally leads to a high nucleation density. Here, the graphene nucleation on the CuNi alloy surfaces is systematically explored and it is revealed that: i) carbon atom dissolution into the CuNi alloy passivates the alloy surface, thereby drastically increasing the graphene nucleation barrier; ii) carbon atom diffusion on the CuNi alloy surface is greatly suppressed by the inhomogeneous atomic structure of the surface; and iii) a prominent increase in the rate of carbon diffusion into the bulk occurs when the Ni composition is higher than the percolation threshold. This study reveals the key mechanism for graphene nucleation on CuNi alloy surfaces and provides a guideline for the catalyst design for the synthesis of graphene and other 2D materials.

Large single‐crystalline graphene wafers are highly desired for realizing many of graphene's potential applications with the optimal performance.^1^ However, the synthesis of wafer‐scale single‐crystalline graphene has been a great challenge in chemical vapor deposition (CVD) growth due to the nucleation of numerous graphene domains on the supported substrate. To achieve growth of single‐crystalline graphene with a large area, it is essential to have very low nucleation densities, which is normally achieved by using very low carbon supply flow.^2^ Cu^3^ and Ni^4^ are the two types of transition metal catalysts that have been explored in most detail for graphene growth, and have shown contrasting mechanisms for graphene growth. Cu has the advantage of enabling growth of single layer graphene due to the self‐limited growth and the extremely low carbon solubility in bulk,[[qv: 2d]] but it suffers from the great drawback of low catalytic activity and consequently has a slow growth rate. In contrast, Ni has superior catalytic power and can boost graphene's growth rate significantly,[[qv: 3b]] but the growth of graphene on Ni substrate is mainly due to poorly controllable precipitation of the dissolved C atoms. Therefore, most graphene samples synthesized on Ni are in nonuniform multilayers. Very recently, we have successfully synthesized inch‐sized single‐crystalline graphene from a single nucleus on a Cu_85_Ni_15_ substrate by using a particular localized carbon feeding strategy over 2.5 h.^5^ A key for the success of this process is the use of a CuNi alloy with a particular Ni concentration (15%), which allows simultaneous achievement of a very fast rate of graphene growth and a very low graphene nucleation density. Compared to Cu, graphene growth on the CuNi alloy is fast due to the presence of abundant C precursors produced by the rapid decomposition of methane on the alloy surface.^5^ Meanwhile, the nucleation density of graphene on the CuNi alloy is greatly reduced despite the higher C concentration. The increased growth rate is attributed to the high catalytic activity of the Ni atoms for the decomposition of CH_4_ molecules,^5^, ^6^ whereas the decreased graphene nucleation density on CuNi alloy has never been understood. According to the classical theory of crystal growth, a high carbon concentration should lead to a high growth rate and a high nucleation density simultaneously.^7^ The findings for the formation of graphene on the CuNi alloy substrate therefore appear completely contrary to the classical theory of crystal growth.

Herein, by combining theoretical calculations with experimental observations, we systematically investigated graphene nucleation on the surface of CuNi alloys with different Ni compositions. It was observed that the nucleation density of graphene decreases monotonically when the Ni composition in the CuNi alloy increases from 0% to 30%. The decreased nucleation density of graphene on CuNi alloy is attributed to two factors: (i) the passivation of the CuNi alloy surface by both nickel and carbon atoms leads to an increased nucleation barrier; and (ii) the Ni content in the alloy increases the barrier for surface diffusion and changes the diffusion behavior of carbon atoms from surface diffusion to the bulk diffusion when the content of Ni in the alloy is higher than the percolation threshold, 19.9%. In addition, the secondary ion mass spectrometry (SIMS) measurements clearly show that more C atoms can be found in the deep bulk of the CuNi alloy when the Ni content of the alloy is increased, supporting our theoretical prediction.

The grain size and nucleation density of graphene on different CuNi alloy surfaces are shown in **Figure** **1**a. Here, the grain size refers to the lateral size of a graphene domain grown on a specific CuNi alloy substrate with a growth time of 10 min. A larger grain size corresponds to a faster graphene growth rate. The graphene domain grown on the Cu_85_Ni_15_ alloy surface has the largest grain size, demonstrating the highest growth rate of graphene on Cu_85_Ni_15_. For alloys with a Ni composition less than 15%, the growth rate of graphene increases with the Ni composition. However, for alloys with Ni content larger than 15%, an increase in the Ni composition leads to reduced grain sizes or slower growth rates. Figure 1b–e shows the optical images of graphene domains obtained on the foils of Cu_95_Ni_5_, Cu_90_Ni_10_, Cu_85_Ni_15_, and Cu_75_Ni_25_ after 10 min of growth at a temperature of 1050 °C. It can be clearly seen that graphene domains grown on Cu_85_Ni_15_ have larger sizes than those grown on Cu_95_Ni_5_ or Cu_90_Ni_10_, while a further increase of Ni content to 25% (Cu_75_Ni_25_) leads to smaller graphene grains. The fast growth of graphene on Cu_85_Ni_15_ can be attributed to the efficient decomposition of CH_4_ on the surface and the extra carbon supply dissolved near the substrate surface, while the slower growth rate at larger Ni content was attributed to the loss of the carbon precursors from the surface through carbon bulk diffusion along the very long Ni chains inside the alloy when the Ni content exceeds the percolation threshold, 19.9%.^5^, ^8^

**Figure 1:**
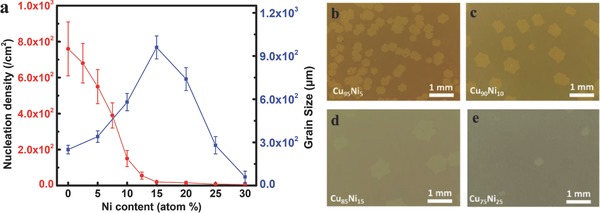
Effects of alloy composition on the nucleation density and domain size of graphene prepared at 1050 °C after growth for 10 min. a) Graphene nucleation density and grain size as a function of Ni content in the CuNi alloy. b–e) Optical images of graphene domains obtained on Cu_95_Ni_5_, Cu_90_Ni_10_, Cu_85_Ni_15_, and Cu_75_Ni_25_ foils under the growth temperature of 1050 °C.

In sharp contrast to the growth rate, the nucleation density of graphene keeps decreasing as the Ni composition increases from 0% to 30% (red line in Figure 1a). Under our experimental conditions, no graphene nucleation was observed on the surface of CuNi alloy with a Ni composition greater than 30%. The decreased graphene nucleation density can be clearly seen from Figure 1b–e, where the number of graphene nuclei decreases as the Ni composition varies from 5% to 25%. It should be noted that concentration of CH_4_ flow can also affect the nucleation density of graphene, but the effect is much smaller compared with the composition of CuNi alloy (Figure S1, Supporting Information). Moreover, the decrease of graphene nucleation density on CuNi alloy with the increase of Ni composition is not dependent on the carbon flow.

Based on the above discussions, it is obvious that the growth rate and nucleation density of graphene show “contradictory” tendencies with the increase of Ni content from 0% to 30%. For Ni content increasing from 0% to 15%, the growth rate of graphene increases, while the nucleation density decreases; whereas during the variation of Ni content from 15% to 30%, both growth rate and nucleation density decrease. To understand such unusual experimental observations, we carried out first‐principle theoretical calculations to explore the key mechanism for the nucleation of graphene on the CuNi alloy surfaces.

The nucleation of graphene starts from the self‐assembly of the carbon precursors (e.g., C atoms) on a substrate surface. To understand the nucleation process, we first calculated the formation energy of a single C atom on CuNi alloy surfaces. The formation energy of a C atom on an alloy surface is defined as

(1) $$E\left(C\right)\;=\;E\left(C@M\right)\;-\;E\left(M\right)\;-\;E_{G}$$

where *E*(C@M) is the energy of a metal substrate with an adsorbed C atom on its surface, *E*(M) is the energy of the metal substrate, and *E*_G_ is the energy of a C atom in graphene.

**Figure** **2** shows the most favorable adsorption sites and the formation energies of a single C atom on CuNi alloy surfaces with Ni composition of 0%, 12.5%, 25%, 50%, 75%, and 100%, respectively. The formation energy of a C atom adsorbed on the Cu(100) surface is 1.98 eV, and it drops to 1.08 eV when the Ni concentration is increased to 12.5%. As the doping concentration reaches 25%, the formation energy of a C atom on the alloy surface is further decreased to 0.61 eV. Notably, the formation energy of a C atom on the alloy surface becomes negative (−0.23 eV) once the Ni concentration is increased to 50%. A negative formation energy of the C atom on an alloy surface means the C atom prefers to stay separated on the alloy surface instead of aggregating into a graphene sheet. Further increase in the Ni content leads to a more negative formation energy of the C atom, e.g., −0.34 and −0.54 eV for 75% and 100% Ni contents, respectively.

**Figure 2:**
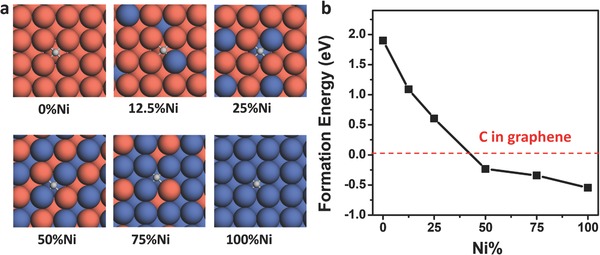
Carbon adsorption on the CuNi alloy surfaces. a) The most favorable adsorption sites of a single C atom on CuNi alloy surfaces with Ni composition of 0%, 12.5%, 25%, 50%, 75%, and 100%, respectively (Cu, orange; Ni, blue; C, gray). b) Formation energies of the adsorbed C atom versus to the Ni composition of CuNi alloys.

The theoretical calculations above demonstrate that the formation of a graphene sheet on the CuNi alloy surface becomes less favorable at a low carbon concentration when the Ni composition is at some point between 25% and 50%. This is consistent with our experimental observation that no nucleation of graphene was found on the CuNi alloy with Ni content larger than 30%.

According to classical 2D nucleation theory, the nucleation rate of graphene can be written as

(2) $$R_{\mathrm{nul}}\;=\;\omega^{*}\Gamma N_{1}\exp\;(-\;G^{*}/kT)$$

where ω* is the attachment rate of C atoms into a cluster of critical size, Γ = [G*/(3πkTN* ^2^)]^1/2^ is the Zeldovich factor, *N*_1_ is the concentration of C atoms, *k* is the Boltzmann constant, *T* is the temperature, and G* is the nucleation energy.^9^ The attachment rate can be estimated as ω* = N*_edge_ *p*(νexp(−*E*_b_/*kT*)), where N*_edge_ is the number of attachment sites of the 2D nucleus. For a sp^2^ network structure, N*_edge_ ∼ (6 × N*)^1/2^, and ν = 10^13^ s^−1^. *E*_b_ is the barrier for attaching a C atom to the graphene, which can be estimated as the diffusion barrier of a C atom on the alloy surface as the graphene edge on the metal substrate is very active. Thus, we can rewrite Equation (2) as following

(3) $$R_{\mathrm{nul}}\;=\;v_{0}N_{1}^{2}{\left(\frac{2G^{*}}{\pi kTN^{*}}\right)}^{\frac{1}{2}}\;\exp\;\left(-\;\frac{E_{b}}{kT}\right)\;\exp\left(-\;\frac{G^{*}}{kT}\right)$$

Therefore, under a fixed growth temperature, the nucleation rate is mainly determined by C atom concentration (*N*_1_), the diffusion barrier *E*_b_, as well as the nucleation barrier G*.

As the graphene nucleation starts from the formation of a carbon cluster, calculating the Gibbs free energy of formation of the carbon cluster is essential to obtain the nucleation barrier of graphene. The Gibbs free energy of formation of the carbon cluster on a metal substrate can be obtained by using the following equation[[qv: 9b]]

(4) $$G\left(C_{N}\right)\;=\;E\left(C_{N}\right)\;-\;N\;\times \;\Delta \mu$$

in which *E*(*C_N_*) is the formation energy of the carbon cluster on the substrate from a C atom in graphene, Δ*µ* is the chemical potential difference of a carbon atom in the feedstock and in the graphene, and *N* is the number of carbon atoms in the cluster. To form a graphene nucleus, the carbon clusters need to be greater than the nucleation size, N*, and their formation energy needs to overcome the nucleation barrier, G*. Based on the crystal nucleation theory, the nucleation barrier and nucleation size of the graphene nucleus can be viewed as the maximum point (N*, G*) of the curve of Gibbs free energy (*G*) versus cluster size (*N*).[[qv: 9a]]

The formation energy, *E*(C*_N_*), of the graphene nucleus under a fixed chemical potential difference Δ*µ* can be obtained by[[qv: 9b]]

(5) $$E\left(C_{N}\right)\;=\;E_{0}\;+\;\surd \left(6N\right)\;\times \;E_{\mathrm{edge}}$$

in which *E*_0_ is a term of constant for a specified metal, it can be regarded as the energy difference between sp^2^ hybridized C in a perfect graphene and in the carbon cluster; *E*_edge_ is the formation energy of each edge atom, and √(6*N*) represents the number of edge atoms.

By replacing *E*(C*_N_*) in Equation (4) with Equation (5), we can get

(6) $$G\left(C_{N}\right)\;=\;E_{0}\;+\;\surd \left(6N\right)\;\times \;E_{\mathrm{edge}}\;-\;N\;\times \;\Delta \mu$$

The nucleation size, N*, and the nucleation barrier, G*, can be obtained by assuming d*G*/d*N* = 0, and thus

(7) $$N^{*}\;=\;\frac{3E_{\mathrm{edge}}^{2}}{2\Delta \mu }$$

(8) $$G^{*}\;=\;E_{0}\;+\;\frac{6E_{\mathrm{edge}}^{2}}{4\Delta \mu }$$

Therefore, the nucleation barrier, G*, on the metal surface is determined by *E*_edge_ under a fixed chemical potential Δ*µ*. According to Equation (5), *E*_edge_ can be obtained by calculating the formation energy of a carbon cluster. Based on this argument, the nucleation of graphene on different CuNi alloy surface can be compared by calculating the formation energy of a carbon cluster.

In this study, a typical carbon cluster, C_24_, is selected. The formation energy of C_24_ on a transition metal surface can be calculated by using a similar definition to that of the single atom given by Equation (1)

(9) $$E\left(C_{\mathrm{24}}\right)\;=\;E\left(C_{\mathrm{24}}@M\right)\;-E\left(M\right)\;-\mathrm{24}\;\times \;E_{G}$$

where *E*(C_24_@M) is the energy of a C_24_ cluster on a metal substrate.

Constrained by the size of the theoretical model, only Cu(100) and three CuNi(100) surfaces with Ni composition of 11%, 16%, and 25% are considered in our calculation. We first studied the formation of a C_24_ cluster on the four pure alloy surfaces (**Figure** **3**a). The formation energy of C_24_ on Cu(100) surface is 13.16 eV, and it decreases to 12.81 eV when the Ni composition of CuNi alloy is 11%. Then, formation energy is further decreased to 11.39 eV when the Ni content is increased to 25%, as shown by the black line in Figure 3c. This is understandable since the Ni—C bond is stronger than the Cu—C bond, and the increase of Ni content on the alloy surface will decrease the formation energy of C_24_. Such an increased reactivity of the alloy surface caused by Ni doping has also been reported in previous studies.^10^

**Figure 3:**
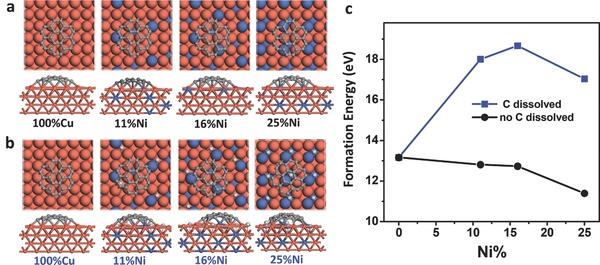
The structure of a C_24_ cluster on CuNi surfaces with and without passivation by carbon atoms. a) Optimized structures of C_24_ on CuNi alloy substrates with different Ni contents (Cu, orange; Ni, blue; C in C_24_, gray; C dissolved in CuNi alloy, light gray). b) Optimized structures of C_24_ on CuNi alloy substrates with dissolved C atoms (CuNi/C). c) The formation energies of C_24_ on CuNi and CuNi/C alloy surfaces.

However, it should be noted that a higher Ni content in the CuNi alloy normally leads to a faster dissolution of carbon atoms and a higher binding energy of the dissociated carbon atoms. Although the dissolution of carbon atoms in Cu is negligible, their solubility in Ni is much higher.^11^ For a CuNi alloy, the solubility of carbon atoms in the subsurface and in the bulk of the substrate should not be neglected. Based on this point, we recalculated the formation energy of C_24_ on CuNi alloy surfaces by taking the effect of dissolved carbon atoms into account. In our calculations, the effect of carbon solubility in CuNi alloy is simulated by adding some additional carbon atoms in the most energetically stable adsorption sites on the alloy surfaces. As shown in Figure 3b, one C atom is added to per surface Ni atom, thus there is a steady increase of dissolved C atoms going from 11%, 16% to 25% Ni alloys. Here, only the dissolution of carbon atoms in the surface layer is considered, since the surface layer is expected to have the largest impact on the graphene nucleation. We denote the CuNi alloy surface with carbon dissolved in it as the CuNi/C surface hereafter. Compared with CuNi alloy surface, the formation energies of C_24_ on the CuNi/C surface were found to be greatly increased (Figure 3c). For example, the formation energies of C_24_ on CuNi/C alloy with Ni composition of 11%, 16%, and 25% are 18.01, 18.67, and 17.04 eV, respectively, which are significantly higher than the formation energy of C_24_ on pure Cu(100) surface (13.16 eV).

The increase of the formation energy can be understood by considering the passivation of the CuNi alloy surface atoms from both the Ni atoms and the dissolved carbon atoms. As shown in Figure S2 (Supporting Information), by replacing the sublayer Cu atoms with Ni atoms, the formation energy of C_24_ increases from 13.16 to 15.26 eV, demonstrating that the doping of Ni content into the Cu bulk could passivate the Cu(100) surface. Another contribution to the surface passivation stems from the dissolved carbon atoms, for example, the formation energy of C_24_ on the (100) surface of Ni_2_C is 6.31 eV higher than that on the Ni(100) surface. This means carbon dissolution can considerably passivate the Ni atoms.

The calculations clearly demonstrate that carbon dissolution into the CuNi alloy can effectively passivate the alloy surface, leading to greatly increased graphene nucleation barriers. It is interesting that the formation energies of C_24_ on the CuNi/C surfaces show a tendency to first rise and then decrease, and thus have a peak at a Ni composition of ≈15%. This demonstrates that the passivation of the CuNi alloy surface has a maximum at a Ni composition of ≈15%, further increase of Ni content makes the alloy surface less passivated.

Above calculation explains very well the decreased nucleation density of graphene on CuNi alloys with Ni composition varying from 0% to 15% because a high nucleation barrier always contributes to a low nucleation density. However, it cannot explain why the nucleation density on Cu_75_Ni_25_ is lower than that on the Cu_85_Ni_15_ surface because the former has a lower nucleation barrier than the latter one.

According to Equation (3), we know that the diffusion of carbon atoms on the surface or in the bulk is another important factor affecting the nucleation rate. Carbon atoms on the alloy surface have two diffusion pathways, one is on the surface and the other is into the bulk of the alloy. To study carbon diffusion, we choose Cu_87.5_Ni_12.5_ and Cu_75_Ni_25_ to represent typical alloys without and with a very long chain of adjacent Ni atoms penetrating into the bulk, respectively. As the percolation threshold of a face centered cubic (fcc) material is ≈19.9%,^8^ alloys with Ni composition less than 19.9% can be roughly represented by the behavior of Cu_87.5_Ni_12.5_, and alloys with Ni composition larger than 19.9% can be represented by that of Cu_75_Ni_25_.

For each alloy substrate, we calculated the diffusion barrier of the C atom on the alloy surface and into the bulk of the alloy. The surface diffusion of a C atom on the surface of Cu_87.5_Ni_12.5_ is shown by the blue line in **Figure** **4**a. It can be seen the highest surface diffusion barrier is 2.44 eV (the energy difference between the highest diffusion barrier and the initial energy), which is higher than that on the Cu(100) surface (1.80 eV as shown in Figure S3 in the Supporting Information). This demonstrates that C diffusion on CuNi alloy surface is slower than that on the pure Cu(100) surface. For a C diffusion into the bulk of Cu_87.5_Ni_12.5_, the calculated highest diffusion barrier is 2.83 eV (red line in Figure 4a). Hence, it can be concluded that C atoms on this alloy surface are likely to diffuse on the surface more than that into the bulk, ensuring sufficient carbon precursor concentrations on the surface of the alloy.

**Figure 4:**
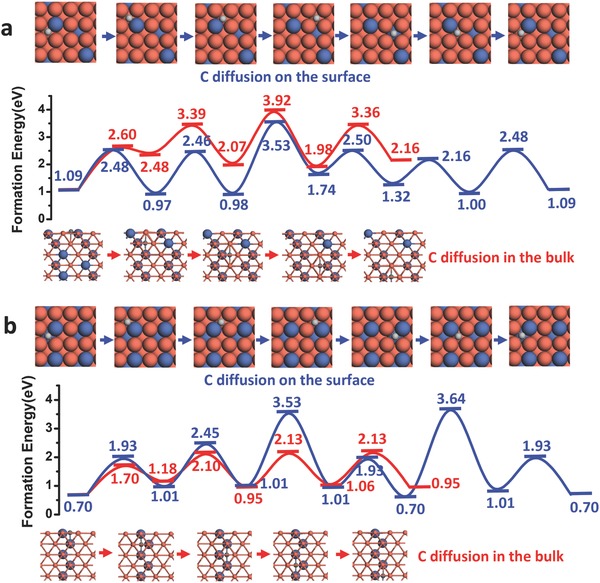
C diffusion on the surface and through the bulk for CuNi alloys. a) C diffusion on the CuNi alloy surface and through the bulk with Ni composition of 12.5%. b) C diffusion on the CuNi alloy surface and through the bulk with Ni composition of 25%.

Although the surface diffusion is very similar, the carbon diffusion into Cu_75_Ni_25_ is quite different from that for Cu_87.5_Ni_12.5_. As a content of 25% Ni results in a concentration that is larger than the percolation threshold of fcc materials, networks of long Ni chains are formed throughout the alloy. Such a Ni chain can assist the diffusion of carbon atoms into the bulk. As shown in Figure 4b, the barrier to surface diffusion (2.83 eV) is similar to that of Cu_87.5_Ni_12.5_ (2.44 eV), while the bulk diffusion has a barrier of 1.43 eV only. This demonstrates that the decomposed carbon atoms on the alloy surface can relatively quickly diffuse into the bulk of the alloy, which will greatly reduce the concentration of the carbon precursor on the surface. Therefore, although the higher Ni composition leads to a relatively fast feedstock decomposition and thus high C concentration,^5^ the effective number of C atoms that participates in the nucleation of graphene will be very low because of the fast diffusion of surface C atoms into the bulk of alloy. This is consistent with the observations in our current experiments as discussed below.

The time of flight (TOF)‐SIMS depth profile analysis is performed to study the C dissolution in CuNi alloys under the same condition of graphene growth procedure. As shown in **Figure** **5**, the measured surface concentrations of C atoms are 10^19^–10^23^ atoms cm^−3^ near the surface of the alloy and this rises sharply with an increase in the relative Ni content. The C atom concentration drops greatly in the bulk of the alloy, indicating that the system is far from thermal equilibrium and the carbon atom concentration inside the bulk was not saturated when the experiments were carried out. In Cu_95_Ni_5_, the C concentration is as low as 10^19^ atoms cm^−3^. Considering that there are ≈10^23^ atoms cm^−3^ in the material, the proportion of the dissolved C in the bulk of Cu_95_Ni_5_ is as low as 10^−4^. This demonstrates that relatively few C atoms have diffused from the surface to the bulk. However, the dissolved C in the bulk of Cu_75_Ni_25_ alloy is ≈10 times higher, demonstrating the faster diffusion of C atoms from the surface to the bulk in this case. The SIMS images of depth profile clearly show that the C dissolution in the Cu_75_Ni_25_ alloy is much higher (with more bright points) than those in other alloys.

**Figure 5:**
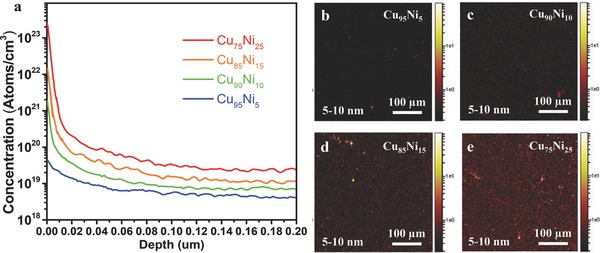
SIMS map of the C concentration on different CuNi alloys. a) The concentration of C atoms in the CuNi alloy with 5%, 10%, 15%, and 25% Ni content after graphene growth by atmospheric pressure CVD (APCVD) at 1050 °C for 15 min. b–e) SIMS depth profile image of C in Cu_95_Ni_5_, Cu_90_Ni_10_, Cu_85_Ni_15_, and Cu_75_Ni_25_, foil at depths between 5 and 10 nm. The color scale represents secondary ion intensity.

Combined the theoretical calculations with the experimental observations, we conclude that the surface diffusion of C atoms dominates for the CuNi alloy with low Ni composition (Ni% < 19.9%) while bulk diffusion of C atoms becomes dominant when the Ni composition of the alloy is higher than the percolation threshold, (Ni% > 19.9%). Based on this argument, we can explain why the nucleation density of graphene on Cu_75_Ni_25_ surface could be lower than that on the Cu_85_Ni_15_. Although graphene on Cu_75_Ni_25_ has lower nucleation barrier and the feedstock can be dissociated faster, the number of C atoms involved in the graphene nucleation is less than that on the Cu_85_Ni_15_ surface because some of the dissociated C atoms diffuse quickly into the bulk. This greatly decreases the graphene nucleation rate and thus leads to a lower nucleation density at high Ni content.

In summary, against our intuition, we have found that the nucleation density of graphene on CuNi alloy surface always decreases with an increase in the Ni content, although systems with a high Ni content have a fast carbon feedstock decomposition. Such a “contradictory” phenomenon has been explained by our theoretical calculations. For CuNi alloy with low Ni composition (<19.9%), the decreased nucleation density can be attributed to the greatly increased nucleation barrier caused by carbon and Ni passivation to the Cu surface, together with the increased diffusion barrier of carbon atoms on the alloy surface. For CuNi alloy with high Ni composition (>19.9%), in addition to the two factors mentioned above, the fast diffusion of surface C atoms into the bulk through the continuous Ni chains in the alloy results in a rapid loss of the surface atoms and which greatly decreases the nucleation density.

## Experimental Section

*Experimental Details—Preparation of CuNi Binary Substrate*: A 6 cm × 6 cm Cu foil (25 µm, 99.8%, Alfa‐Aesar) was first electrochemically polished with a current density of ≈0.3 A cm^−2^ for 90 s, then annealed at 1050 °C in a mixture of Ar/H_2_ (400/100 sccm) for 2 h followed by electroplated with a current density of ≈0.01 A cm^−2^. A Ni film with a certain thickness was deposited on the Cu foil at a rate of 200 nm min^−1^. The polishing solution was a mixture of 500 mL of water, 250 mL of ethanol, 250 mL of orthophosphoric acid, 50 mL of isopropyl alcohol, and 5 g of urea. The electrolytic solution consisted of 1 L of water, 280 g of Ni_S_O_4_⋅6H_2_O, 8 g of NiCl_2_⋅6H_2_O, 4 g of NaF, and 30 g of H_3_BO_3_.

*Experimental Details—Characterization of CuNi Surface*: X‐ray diffraction (XRD) and electron backscattered diffraction (EBSD) are employed to analyze the crystallinity and surface morphology of metal substrate. The XRD spectra shows that grains in the CuNi foil have a wide range of crystallographic orientations before annealing but a strong (100) texture is developed after high temperature annealing (Figure S4a, Supporting Information). It could also be observed that Cu and Ni were completely interdiffused to each other to form a uniform CuNi alloy. However, the density of the grain boundaries did not increase obviously with the increasing Ni content. The EBSD image showed that the average grain size on Cu_95_Ni_5_, Cu_85_Ni_15_ and Cu_75_Ni_25_ surfaces was ≈200–500 µm (Figure S4b–d, Supporting Information). Longer pregrowth annealing under Ar/H_2_ flow could help in the production of CuNi (100) facets while mitigating surface roughness and grain boundary (GB) migration. The uniform orientation of CuNi alloy after polishing and annealing is helpful to improve the nucleation density and quality of single crystalline graphene.

*Experimental Details—Graphene Growth*: CuNi alloy foils containing 5–15 at% Ni (Cu_95_Ni_5_, Cu_90_Ni_10_, Cu_85_Ni_15_, Cu_75_Ni_25_, and Cu_70_Ni_30_) were used as substrates. Prior to growth, the CuNi foils were cleaned by hydrochloric acid, acetone, isopropanol, and deionized water to remove the surface oxide and organic impurity. The CuNi substrate was then loaded into a quartz tube with a diameter of 50 mm. The substrates were annealed at 1050 °C 2 h in a H_2_ and Ar flow (H_2_/Ar = 50/1000 sccm) at atmosphere pressure to further increase its surface flatness and grain size. Then, 80–120 sccm methane (0.5% CH_4_ diluted in Ar), 15 sccm H_2_, and 300 sccm Ar were fed into the CVD system. Millimeter‐sized graphene domains could be obtained even after exposure to methane for only about ≈5–10 min. After that, the substrates cooled down to room temperature in a mixed Ar/H_2_ flow.

*Experimental Details—SIMS Measurements*: SIMS can provide detailed chemical information of the surface and subsurface of a material with high‐accuracy depth profiling. In this study, TOF‐SIMS (ION‐TOF GmbH TOF.SIMS5) depth profile analysis was performed to further test the component of alloys and the carbon dissolution in CuNi alloys. It could be observed that Cu and Ni were completely interdiffused to each other to form CuNi alloy foils with the atomic proportions determined by the amount of the deposited Ni film at high temperature annealing. The SIMS measurement provided more decisive information to understand the local nucleation feature and growth conditions on CuNi alloy. Surface enrichment of carbon was detected on the CuNi alloy after the substrate was exposed to methane for few minutes. The surface concentration of carbon reached 1 × 10^22^–1 × 10^23^ atoms cm^−3^ and raised with the increasing of the proportion of Ni content (see Figure 5 in the main text). However, the carbon concentration was far below the saturation state in the bulk.

*Computational Details*: For the calculation of binding energy of monolayer graphene on the substrate, a supercell with a seven‐layer Ni (or Cu) slab and a vacuum layer of 15 Å were used along the *z* direction (normal to the surface). Also, a four‐layer Ni (or Cu) slab with the same vacuum was used to calculate the formation energy of C_24_ on the substrate.

Density functional theory (DFT) calculations were performed using the Vienna Ab initio Simulation Package,^12^ and the projector‐augmented‐wave method was applied^13^ with the Perdew–Burke–Ernzerhof (PBE)^14^ generalized gradient approximation functional. To treat the van der Waals interactions between graphene and the substrate, the adsorption of graphene on substrate was modeled using the widely used dispersion‐corrected DFT‐D_2_ of the PBE functional.^15^ The climbing‐image nudged elastic band method^16^ was exploited to locate the transition states of C diffusion on the substrate surface and into the bulk of the alloy. A Monkhorst–Pack scheme was applied with 2 × 2 × 1 *k*‐points selected. A plane‐wave basis kinetic energy cut‐off of 400 eV was used in all the calculations.

## Conflict of Interest

The authors declare no conflict of interest.

## Supporting information

Supplementary
