# Supplementary material for: How Low Nucleation Density of Graphene on CuNi Alloy is Achieved
Source: Adv Sci (Weinh). 2018 Mar 12;5(6):1700961. doi: 10.1002/advs.201700961 (PMC6010776; doi:10.1002/advs.201700961)
Supplement: Supplementary file 1 — Supplementary [file ADVS-5-1700961-s001.pdf]

Copyright WILEY-VCH Verlag GmbH & Co. KGaA, 69469 Weinheim, Germany, 2018.

ADVANCED SCIENCE
Open Access

Supporting Information

for *Adv. Sci.,* DOI: 10.1002/advs.201700961

How Low Nucleation Density of Graphene on CuNi Alloy is Achieved

Yifan Liu, Tianru Wu, Yuling Yin, Xuefu Zhang, Qingkai Yu, Debra J. Searles, Feng Ding, Qinghong Yuan, and Xiaoming Xie**

Copyright WILEY-VCH Verlag GmbH & Co. KGaA, 69469 Weinheim, Germany, 2016.

Supporting Information

How low nucleation density of graphene on CuNi alloy is achieved

Yifan Liu, Tianru Wu, Yuling Yin, Xuefu Zhang, Qingkai Yu, Debra J. Searles, Feng Ding, Qinghong Yuan, Xiaoming Xie**

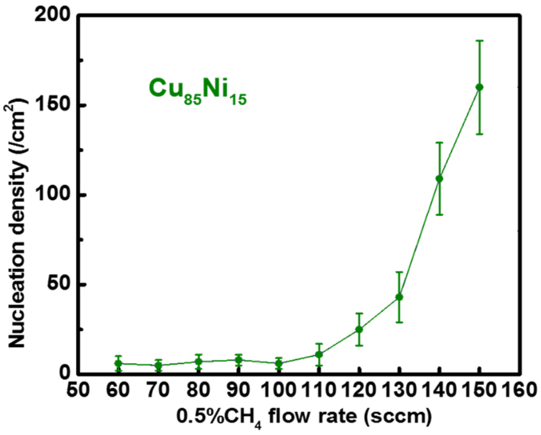

Supplementary Figure 1 Graphene nucleation density as a function of carbon precursor flow rate on $Cu_{85}Ni_{15}$ under the growth temperature of 1050 °C.

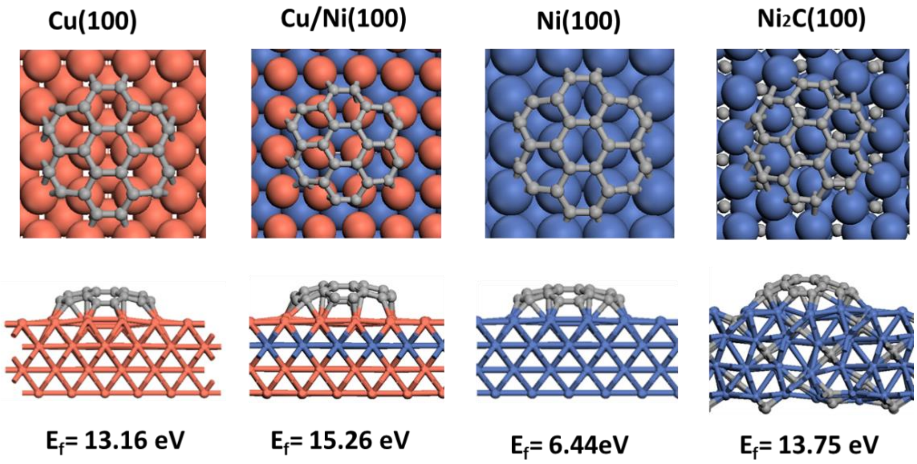

Supplementary Figure 2 The formation energies of C_{24} on Cu(100), Cu/Ni(100), Ni(100) and Ni_2C(100) surfaces.

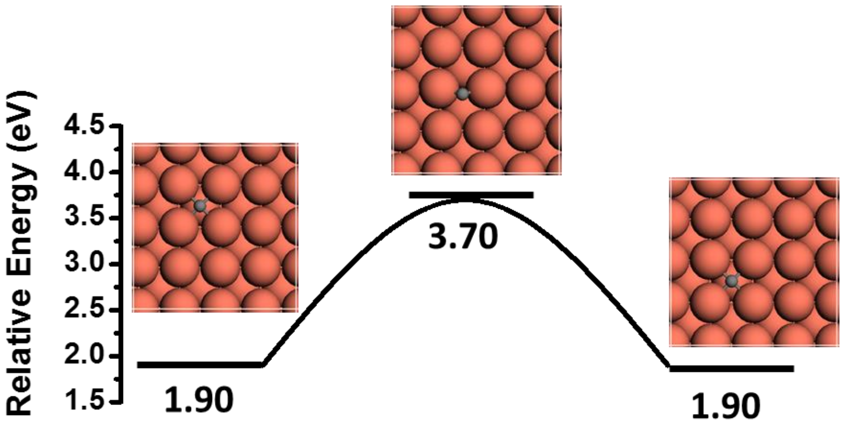

Supplementary Figure 3 The energy curve of C diffusion on Cu(100) surface.

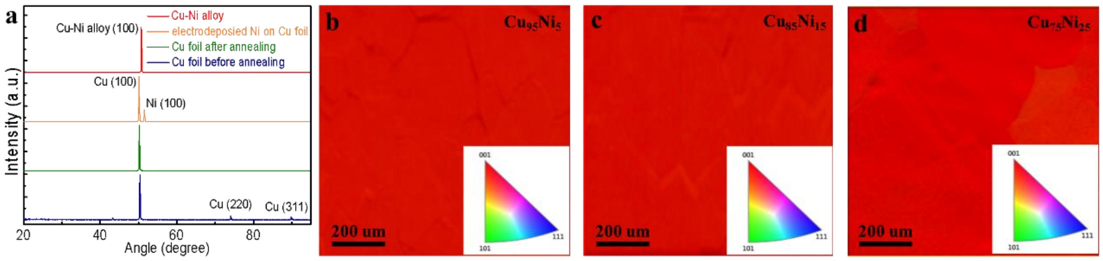

Supplementary Figure 4 a) XRD spectra of Cu and Cu-Ni bilayer before and after the annealing process. **b-d)** EBSD image of $Cu_{95}Ni_5$, $Cu_{85}Ni_{15}$ and $Cu_{75}Ni_{25}$ grains after high temperature annealing
